# Em Busca do Marcador Perfeito

**DOI:** 10.36660/abc.20230193

**Published:** 2023-04-18

**Authors:** Luiz Maurino Abreu

**Affiliations:** 1 Estimulocor – Avaliação Clínica Cardiológica Rio de Janeiro RJ Brasil Estimulocor – Avaliação Clínica Cardiológica, Rio de Janeiro, RJ – Brasil

**Keywords:** Biomarcador, Doença Arterial Coronariana/prevenção e controle, Imunidade, Oncostatina M, Glicoproteína 130

As doenças cardiovasculares são responsáveis pela maior parte da morbimortalidade contemporânea. No Brasil, dados mostram crescimento dos indicadores com grande impacto socioeconômico.^1^

Fatores de risco como lipídios alterados, hipertensão, tabagismo, diabetes, erros alimentares, fatores psicossociais e inatividade física são responsáveis pela maior parte do risco de infarto do miocárdio em todo o mundo em ambos os sexos e em todas as idades em todas as regiões, como foi bem demonstrado no Estudo INTERHEART.^2^

A fisiopatologia da doença coronariana ainda gera muitas dúvidas, e diversos estudos realizados ao redor do mundo buscam esclarecer o mecanismo e os fatores relacionados ao aparecimento e progressão da doença aterosclerótica, com a descrição de diversos atores, muitos dos quais também estão envolvidos em outras doenças, cuja base está na atividade inflamatória.^3^

As citocinas, proteínas que modulam a função de outras células ou da célula que as gerou, são apontadas como potenciais causadoras, protetoras ou regeneradoras da agressão do processo aterosclerótico. A oncostatina M, uma citocina membro da família IL-6, está presente em diversas situações clínicas, assim como seu receptor (sOSMR) e a glicoproteína 130 (sgp130).^4^

No artigo dos Arquivos Brasileiros de Cardiologia,^5^ os autores estudaram o comportamento desses fatores no contexto da doença coronariana aterosclerótica. Realizam uma elegante análise exploratória transversal, em pacientes com critérios de DAC (CHD e ACS) e um grupo controle sem doença clinicamente detectada, do comportamento sérico de OSM, sOSM e gpt 130, que mostrou que pacientes com DAC tiveram significativamente níveis mais baixos de sOSMR e sgp130 e valores mais altos de OSM em comparação aos controles, e com variações relacionadas à idade, sexo, hipertensão e alguns medicamentos.^5^

O termo biomarcador traz o significado de um achado em exames funcionais ou de imagem que denuncia alguma alteração da normalidade, que pode representar uma doença ou uma condição de risco para o seu aparecimento. Na medicina, os biomarcadores são aqueles que são detectáveis no sangue ou nas secreções.

Devido à sua importância epidemiológica, a doença cardiovascular estimula a busca por marcadores diagnósticos e prognósticos. Inúmeras variáveis são estudadas, muitas das quais já estão incorporadas aos escores de risco. Por exemplo, epidemiologia, exposição a fatores externos, como tipo de alimentação, nível de sedentarismo, tabagismo e outras drogas, além de fatores socioeconômicos e climáticos.

Uma análise crítica da pesquisa básica em fisiopatologia é essencial para gerar hipóteses e identificar a complexidade do problema e sua aplicabilidade à prática médica. O que podemos esperar de um biomarcador à luz do que já sabemos? O ideal seria uma medição fácil e rápida, com um custo adequado e proporcional ao seu impacto, superando os métodos e parâmetros existentes. Só assim um novo biomarcador pode ajudar na melhor tomada de decisão em relação a uma abordagem médica.

Objetivamente, incorporar um novo biomarcador aos que já utilizamos classicamente exige que ele ofereça informações adicionais aos já conhecidos. Para tanto, duas técnicas de análise são utilizadas em estudos prospectivos: a estatística C e o Índice de Reclassificação Neto (RN). No primeiro método, a estatística C, é possível determinar o potencial de discriminação com o uso do biomarcador entre quem vai e quem não vai apresentar o desfecho, enquanto o RN busca avaliar o quanto o novo biomarcador pode reclassificar indivíduos para maior risco ou menor.^6^

Embora ainda haja muito a ser esclarecido sobre o papel de OSM, sOSMR e sgp 130 na DAC, estudos preliminares sugerem que eles podem ser úteis na identificação de pacientes com risco aumentado de eventos cardiovasculares e na avaliação da progressão da doença.^7^ Entretanto, mais pesquisas são necessárias para compreender plenamente seu papel na DAC e como podem ser utilizados na prática clínica, devendo esclarecer diversos aspectos dos efeitos antagônicos de proteção ou agravamento da doença coronariana, conforme descrito na literatura.^8 , 9^

A medicina personalizada é um conceito que todos os médicos devem esforçar-se por oferecer aos seus doentes, tendo em conta que se trata de indivíduos com necessidades e padrões de doença únicos. A tecnologia nos traz novas ferramentas para personalizar o cuidado cardiovascular, e sua perspectiva sobre esse cuidado é infinita. Dispositivos móveis com sensores capazes de medir e transmitir informações saem do campo da ficção e já permitem a medição precisa e contínua de dados biológicos, registrados e analisados, por meio de aplicativos que transmitem as informações. As novas tecnologias têm o potencial de revolucionar a prevenção e o gerenciamento de doenças.^10^

Entretanto, reforçamos com nossos pacientes que um estilo de vida saudável, com alimentação adequada, atividade física e controle dos fatores clássicos, é um grande marcador de boa evolução para uma vida saudável.
