# Supplementary material for: Em Busca do Marcador Perfeito
Source: Arq Bras Cardiol. 2023 Apr 18;120(4):e20230193. [Article in Portuguese] doi: 10.36660/abc.20230193 (PMC10263418; doi:10.36660/abc.20230193)
Supplement: Translation

# Translation: In Search of the Perfect Marker

**Authors:** Luiz Maurino Abreu

**Affiliations:** 1 Brasil Estimulocor – Avaliação Clínica Cardiológica, Rio de Janeiro, RJ – Brasil

**Keywords:** Byomarkers, Coronary Artery Disease/prevention and control, Immunity, Oncostatin-M, Glycoprotein 130, Atherosclerosis

Cardiovascular diseases are responsible for most contemporary morbidity and mortality. In Brazil, data show growth in indicators with a great socioeconomic impact.^1^

Risk factors such as altered lipids, hypertension, smoking, diabetes, dietary errors, psychosocial factors, and physical inactivity are responsible for most of the risk of myocardial infarction worldwide in both sexes and at all ages in all regions, as was well demonstrated in the INTERHEART Study.^2^

The pathophysiology of coronary disease still raises many doubts, and several studies carried out around the world seek to clarify the mechanism and factors related to the appearance and progression of atherosclerotic disease, with the description of several actors, many of whom are also involved in other diseases, whose basis is in inflammatory activity.^3^

Cytokines, which are proteins that modulate the function of other cells or the cell that generated them, are identified as potential causes, protectors, or regenerators of the aggression of the atherosclerotic process. Oncostatin M, a cytokine member of the IL-6 family, is present in several clinical situations, as well as its receptor (sOSMR) and glycoprotein 130 (sgp130).^4^

In the article from *Arquivos Brasileiros de Cardiologia* ,^5^the authors studied the behavior of these factors in the context of atherosclerotic coronary disease. It performs an elegant cross-sectional exploratory analysis, in patients with CAD criteria (CHD and ACS) and a control group without clinically detected disease, of the serum behavior of OSM, sOSM, and gpt 130, which showed that patients with CAD had significantly lower levels of sOSMR and sgp130 and higher OSM values compared to controls, and with variations related to age, gender, hypertension, and some medications.^5^

The term biomarker brings the meaning of a finding in functional tests or imaging denouncing some change in normality, which may represent a disease or a risk condition for its appearance. In medicine, biomarkers are those that are detectable in blood or secretions.

Due to its epidemiological importance, cardiovascular disease encourages the search for diagnostic and prognostic markers. Countless variables are studied, many of which have already been incorporated into risk scores, for instance, epidemiology, exposure to external factors, such as type of diet, level of a sedentary lifestyle, smoking, and other drugs, and socioeconomic and climate factors.

A critical analysis of basic research in pathophysiology is essential to generate hypotheses and identify the complexity of the problem and its applicability to medical practice. What can we expect from a biomarker in light of what we already know? The ideal would be easy and quick measurement, with an adequate cost proportional to its impact, surpassing the existing methods and parameters. This is the only way a new biomarker could help in better decision-making regarding a medical approach.

Objectively, incorporating a new biomarker into those we already use classically requires it to offer additional information to those already known. For this purpose, two analysis techniques are used in prospective studies: the C statistic and the Net Reclassification Index (NR). In the first method, the C statistic, it is possible to determine the potential for discrimination using the biomarker between those who will and those who will not present the outcome. At the same time, the NR seeks to assess how much the new biomarker can reclassify individuals for greater risk or less.^6^

Although much remains to be clarified about the role of OSM, sOSMR, and sgp 130 in CAD, preliminary studies suggest they may be useful in identifying patients at increased risk of cardiovascular events and assessing disease progression.^7^More research is necessary to fully understand their role in CAD and how they can be used in clinical practice. It should clarify several aspects of antagonistic effects of protection or worsening of coronary disease, as described in the literature.^8 , 9^

Personalized medicine is a concept that all physicians should strive to offer their patients, keeping in mind that these are individuals with unique needs and disease patterns. Technology brings us new tools to personalize care, and its perspective on cardiovascular care is endless. Mobile devices with sensors capable of measuring and transmitting information leave the field of fiction and already allow the precise and continuous measurement of biological data, recorded and analyzed, using applications that transmit the information. New technologies have the potential to revolutionize disease prevention and management.^10^

Meanwhile, let us reinforce with our patients that a healthy lifestyle, with proper nutrition, physical activity, and control of classic factors, is a great marker of good evolution for a healthy life.
